# Malignant Hip Flexion Failure Syndrome: An Oncologic Disease Compared to Malignant Psoas Syndrome

**DOI:** 10.7759/cureus.67343

**Published:** 2024-08-20

**Authors:** Yojiro Ishikawa, Satoshi Teramura, Kengo Ito, Takayuki Yamada

**Affiliations:** 1 Radiology, Tohoku Medical and Pharmaceutical University, Sendai, JPN

**Keywords:** iliopsosa muscle, psosa major muscle, malignant lumbosacral plexopathy, malignant hip flexion failure, malignant psosa syndrome

## Abstract

Malignant psoas syndrome (MPS) causes painful hip immobilization when a malignant tumor reaches the psoas muscle. However, there exists a different condition in which a malignant tumor invades the psoas muscle, leading to hip flexion failure without painful hip immobilization. This study aimed to define malignant hip flexion failure syndrome (MHFFS) as tumors located in the upper lumbar region or at the lesser trochanter of the femur, near the origin or termination of the psoas muscle, and to compare its prevalence, characteristics, and outcomes with those of classical MPS. We analyzed 291 patients who received palliative radiotherapy (RT) in the lumbar, pelvic, and lower leg regions from 2013 to 2023. The prevalence of MPS and MHFFS, pathological features, distinctive clinical presentations, treatment modalities, and treatment outcomes have been summarized. We also defined the 'Clinical sign reported by Ishikawa and Teramura (IT sign)' to describe the characteristic action of lifting the affected lower leg with both hands in MHFFS cases and assessed its clinical significance. Among the 291 patients, 6 (2.1%) had MHFFS and 11 (3.8%) had MPS. MHFFS resulted from metastatic tumors in the 11th and 12th thoracic vertebrae, as well as the 1st and 2nd lumbar vertebrae or the lesser trochanter of the femur, and it was characterized by hip and groin pain along with hip flexion dysfunction. All cases showed a positive IT sign. The response to RT varied, with symptomatic improvement observed in 50% of the patients. MPS is characterized by tumor invasion of the psoas muscle, causing severe lumbosacral nerve pain. Strong opioids were used for pain management in all patients, and epidural anesthesia was required in some patients. The median survival time of patients with MPS and MHFFS was 13.2 months. MPS required opioids more potently than MHFFS, but MHFFS responded relatively well to early RT.

## Introduction

A condition characterized by malignant tumor invasion of the psoas muscle is known as malignant psoas syndrome (MPS). It is caused by direct invasion of the psoas muscle by metastatic disease or primary malignancy [1-10]. Although reports on MPS are limited and the exact pathogenesis of MPS remains incompletely understood, MPS occurs when there is imaging or pathological evidence of malignant disease within the psoas major muscle on the affected side. This condition results in hip flexion immobilization, with pain intensified during hip extension, and lumbosacral plexopathy, which affects the 1st-4th lumbar vertebrae. Physical examination often reveals neuropathy in the 1st-4th nerve roots on the affected side and suggests spasm of the psoas major muscle [2-4]. Moreover, as these lesions progress, they may extend into the spinal canal, causing epidural compression of the spinal cord or cauda equina, which can lead to severe complications [3,11].

Teramura S et al. and Ishikawa Y et al. reported one case each of conditions resembling MPS but with different clinical presentations [12,13]. In these reports, the tumors invaded the psoas muscle, leading to lumbar back, hip, or groin pain. However, unlike typical MPS, there was no painful hip immobilization or weakness of the psoas muscle (hip flexion failure). Both patients experienced mobility impairment and depended on wheelchairs, walkers, or canes because of insufficient hip flexion. However, they did not experience worsening pain during hip extension, a characteristic feature of MPS. We have named this distinctive symptomatic condition malignant hip flexion failure syndrome (MHFFS).

In summary, while MPS is characterized by an inability to extend the hip due to pain and spasm of the psoas major muscle, MHFFS is characterized by an inability to flex the hip without the associated pain during hip extension. In this study, we investigated the mechanical differences between MHFFS and typical MPS in terms of frequency, imaging findings, symptoms, treatment options, and response to treatment.

## Materials and methods

Patients

**Figure 1:**
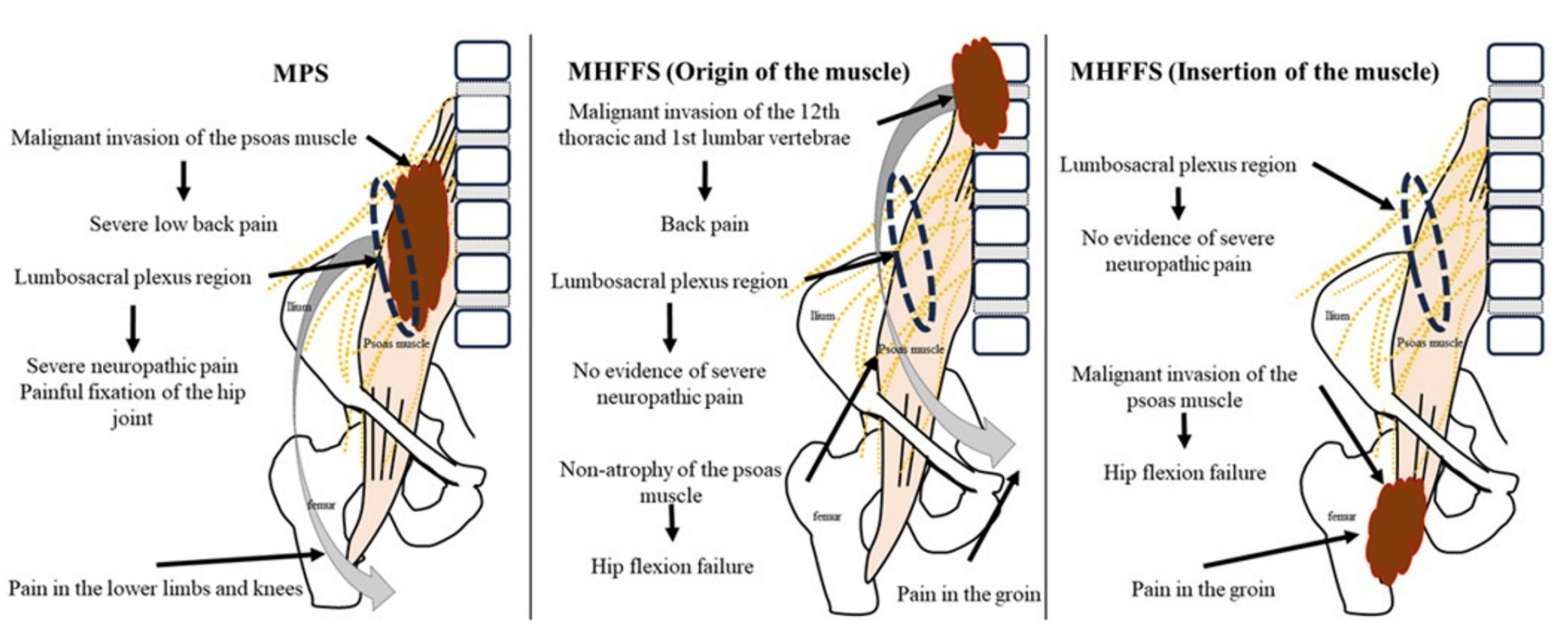
Differentiating malignant psoas syndrome (MPS) and malignant hip flexion failure syndrome (MHFFS): clinical characteristics and disease localization. MPS: The tumor is located within the belly of the psoas muscle and infiltrates the lumbosacral plexus, inducing intense pain. Characteristic symptoms include pain in the lower extremities and knees, potentially arising from the femoral nerve. Pain is intensified with hip extension. The psoas test yields positive results, manifesting as hip immobilization due to pain, often leaving the patient in a sitting or side-lying position, with challenges in assuming a supine position. MHFFS (Origin of the muscle): Originating from the lower thoracic and upper lumbar spine, a tumor typically extends to the cephalic end of the psoas muscle. This often results from a tumor near the lower thoracic spine, such as at the 12th thoracic vertebra, or the upper lumbar spine, such as at the 1st and 2nd lumbar vertebrae. Unlike MPS, there is no tumor in the belly of the psoas muscle, nor is there pain originating from the lumbosacral plexus. The psoas muscle test yields negative results, and there is no painful immobilization of the hip. Patients may report pain in the groin or near the hip due to impairment at the 12th thoracic and the 1st and 2nd lumbar spinal cord levels. MHFFS (Insertion of the muscle): The tumor extends to the distal end of the psoas muscle, often leading to involvement of the psoas muscle and pathological fracture of the lesser trochanter. Unlike MPS, this does not cause pain originating from the lumbosacral plexus due to the absence of tumors in the belly of the psoas muscle. The psoas muscle test yields negative results, and there is no pain associated with hip immobilization. MPS: Malignant psoas syndrome; MHFFS: Malignant hip flexion failure syndrome. Image credits: Yojiro Ishikawa.

From January 2013 to December 2023, 440 patients underwent palliative radiation therapy (RT) for primary tumors, bone metastases, or lymph node metastases at our institution. In this study, 291 patients with malignancies who underwent palliative RT for pain control related to primary malignancy or metastatic tumors in the hip, pelvis, or proximal femur exhibited clinical characteristics associated with hip flexion dysfunction. These patients were categorized into two groups: MHFFS and MPS (Figure 1). Information on their prevalence, clinical characteristics, response to RT, and overall survival will be presented. Additionally, we will showcase three specific cases in the MHFFS group through case reports. This study involved a single-center retrospective review of clinical records at our institution during the same period. This retrospective study complied with the Declaration of Helsinki, and our institution’s institutional review board approved the study (approval number: 20232085). All eligible patients provided written informed consent before treatment. Each lesion was diagnosed by radiologists or radiation oncologists.

Eligibility criteria

MHFFS

**Figure 2:**
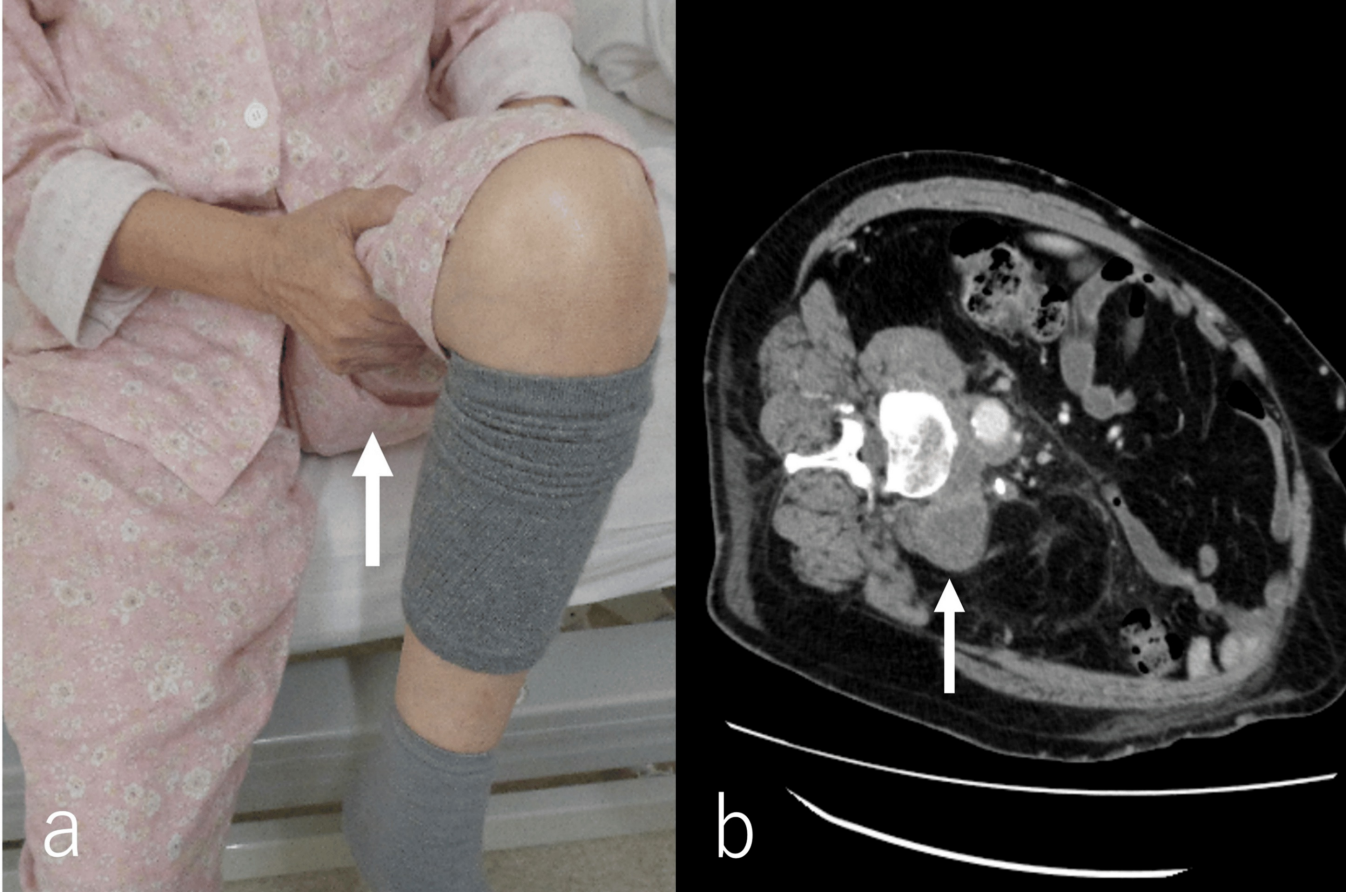
Demonstration of the Ishikawa-Teramura sign (IT sign) and CT imaging in malignant psoas syndrome (MPS). The Ishikawa-Teramura sign (IT sign), as described by Ishikawa et al. and Teramura et al., is employed to assist hip flexion by lifting the lower extremity with the patient’s own hand when hip flexion is insufficient [12,13]. This sign is characterized by a specific movement where the patient uses their hand to assist in raising the leg, particularly during activities such as wheelchair transfers. Patients sometimes report pain near the hip but rarely during hip flexion (a, arrow). The contrast-enhanced CT image illustrates malignant psoas syndrome (MPS). In some instances, the pain prohibits patients from lying supine, requiring CT imaging to be conducted in a lateral decubitus position (b, arrow).

To distinguish from MPS, the following patients were included in the MHFFS group based on characteristics described in previous case reports [12,13]: (1) patients with pain or gait disorders originating from malignancies in the lumbar, pelvic, hip (including groin), or proximal femur region; (2) patients with a pathologically or clinically confirmed primary tumor diagnosis; (3) patients diagnosed by an orthopedic surgeon, neurologist, or experienced radiation oncologist; and (4) patients who displayed clinical evidence of hip flexion failure caused by malignancies in the psoas muscle or at a location proximal to the psoas muscle. Clinical examinations for MHFFS included observations of patients displaying a lifting motion of the affected lower extremity during wheelchair transfers, a sign previously described by Teramura S et al. and Ishikawa Y et al. (Ishikawa-Teramura sign (IT sign)) [12,13]. Patient complaints, such as difficulty in lifting the affected lower extremity while exiting a car or inability to wear certain footwear due to failure to lift the lower extremity, were also considered part of the IT sign. One of the criteria was whether the patient exhibited no muscle weakness in the lower extremities and flexed the hip joint during knee flexion while in the supine position (a condition in which flexion of the knee causes the hip joint to flex in a manner resembling normal flexion). Typical IT signs are depicted in Figure 2a. Patients in the MHFFS group could lie supine without experiencing painful fixed flexion of the ipsilateral hip, and there was no exacerbation of pain with attempted hip extension (negative psoas test).

MPS

Based on the characteristics described in previous reports [2-4], the inclusion of patients in the MPS group was based on the following criteria: (1) patients with pain or gait disorder of malignant origin in the lumbar, pelvic, or hip (including groin), (2) a pathologically or clinically confirmed diagnosis of a primary tumor, (3) clinical evidence of proximal lumbosacral (L1-4) plexus involvement and painful hip immobilization of the ipsilateral hip, and (4) worsening of pain with attempted hip extension (positive psoas test). Worsening pain with attempted hip extension was also indicated by the inability of the patient to lie in the supine position or to be placed in the side-lying position for CT examinations. A typical image is shown in Figure 2b.

Exclusion criteria

Based on the characteristics described in previous case reports [12,13], cases that involved RT only to the cervical to thoracic region (up to the 10th thoracic vertebra), ribs, sternum, chest wall, distal femur, skull, collarbone, shoulder blade, shoulder, and upper limbs were excluded. Additionally, cases with inadequate electronic medical records were also excluded.

Radiation therapy

The study included 17 patients who received RT at our hospital. All plans were for 3D conformal RT (3DCRT). Each plan was generated using the RayStation treatment planning system (RaySearch Laboratories AB, Stockholm, Sweden). All RT plans used a 4, 6, or 10 MV X-ray beam on a Synergy linear accelerator (Elekta, Stockholm, Sweden).

Statistical analyses

Overall survival (OS) was measured from the start of 3DCRT and calculated as OS after treatment, including both MHFFS and MPS cases. Statistical analyses were performed using JMP v16 (SAS Institute Inc., Cary, NC, USA).

Follow-up evaluation after treatment

The follow-up intervals in our study were determined based on the medical records available from the retrospective analysis. Follow-up evaluations were performed at 1- to 3-month intervals. However, this decision was left to the discretion of the attending physician.

## Results

Prevalence and distribution of MPS and MHFFS among cancer types and irradiation sites

Of the 291 patients included in the study, six (2.1%) had MHFFS, and eleven (3.8%) had MPS. The most common cancer was lung cancer (72 patients, 24.7%), followed by breast cancer (28 patients, 9.6%) and prostate cancer (27 patients, 9.3%). Additionally, 24 patients had rectal and stomach cancers (8.3% each), 21 patients (7.2%) had colon cancer, and 14 patients (4.8%) had uterine cancer. Ten patients each (3.4%) had renal and bladder cancers. Sixty-one patients (21.0%) presented with other types of cancers. Irradiation was performed for the lumbar spine in 92 patients (31.6%), lower thoracic spine in 33 patients (11.3%), pelvis in 61 patients (21.0%), femur in 35 patients (12.0%), sacrum in 24 patients (8.3%), and pelvic lymph nodes, organs, and soft tissue in 46 patients (15.8%).

MHFFS

The characteristics of patients with MHFFS are summarized in Table 1. The median age of the patients was 70 years (range: 76-89 years). The primary diseases were liver and breast cancers in two patients each. Among the six cases, four were diagnosed by MRI, although contrast-enhanced MRI was not performed. The metastases responsible for MHFFS were located either in the upper lumbar region or the lesser trochanter of the femur near the origin or termination of the psoas muscle. There were no cases of direct invasion from the primary lesion; all were metastatic. The median tumor size was 3.5 cm (range from 1.1 cm to 7.6 cm), with larger tumors observed in the lesser trochanter. The IT sign was positive in all cases, with four cases confirmed through actual examination and two cases documented in the medical records based on the IT sign. The symptoms included back pain, hip pain, or both, with no signs of femoral nerve involvement, such as numbness in the lower legs or knees or weakness in the lower extremities. RT was administered at a palliative dose in all patients, with three patients experiencing improved hip flexion during and immediately after RT.

**Table 1:** Summary of clinical features of malignant hip flexion failure syndrome cases. AD: Adenocarcinoma; Gy: Gray; HCC: Hepatocellular carcinoma; IDC: Invasive ductal carcinoma; IT sign: Ishikawa-Teramura sign; MHFFS: Malignant Hip Flexion Failure Syndrome; PS: Performance Status; S-colon: Sigmoid colon.

	Age (years)	Sex	Primary site	Pathology	Imaging	Cause of MHFFS	Maximum lesion diameter (cm)	PS	Clinical symptom	Gait disorder	TI sign	Radiation regimen	Treatment Response
Case 1	89	M	Liver	HCC	CT/MRI	Metastasis	4	2	Lower limb, hip and low back pain	Yes	Positive	25	Unclear
Case 2	72	F	Breast	IDC	CT/MRI	Metastasis	1.1	1	Lower limb, hip and low back pain	Yes	Positive	37.5	Well
Case 3	75	F	Breast	IDC	CT/MRI	Metastasis	2.2	1	Lower back pain	Yes	Positive	36	Unclear
Case 4	71	M	Lung	AD	CT	Metastasis	7.6	2	Lower limb and hip pain	Yes	Positive	30	Unclear
Case 5	75	M	Liver	HCC	CT	Metastasis	5.2	2	Lower limb and hip pain	Yes	Positive	20	Well
Case 6	76	F	S-colon	AD	CT/MRI	Metastasis	1	2	Hip pain	Yes	Positive	30	Well

Case 1

**Figure 3:**
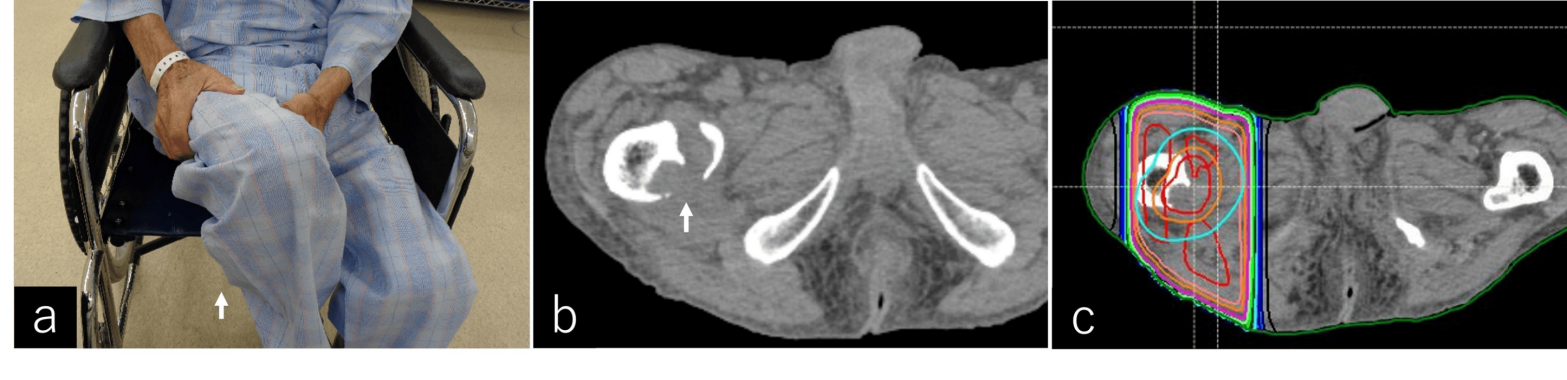
Case 1: Ishikawa-Teramura sign, CT imaging of metastatic mass, and radiation therapy planning. Ishikawa-Teramura Sign (IT sign) (a). A simple CT scan of the pelvic region revealed a metastatic mass and a pathological fracture at the right lesser trochanter of the femur (arrow) (b). Radiation therapy (RT) planning using RayStation (RaySearch Laboratories AB, Stockholm, Sweden) involved irradiation from two fields in the anterior-posterior direction. The patient received 25 Gy in 5 fractions of RT for the right femoral lesser trochanter (c).

An 89-year-old man presented with back and right hip pain. The clinical diagnosis was lung metastasis and multiple bone metastases, including the 4th rib, 6th thoracic transverse process, and right femur lesser trochanter, originating from primary liver cancer. However, because of the patient’s advanced age, conservative treatment was chosen. Six months after his initial visit, the patient presented to the emergency room with pain in his right hip after carrying a load and difficulty walking. During the physical examination before RT, the patient experienced difficulty flexing his right hip when transitioning from a wheelchair and was observed holding his right lower extremity elevated by both hands (positive IT sign) (Figure 3a). He was diagnosed with a fracture of the right femoral lesser trochanter on CT (Figure 3b), and a palliative RT plan was devised (Figure 3c). The patient received 25 Gy in 5 fractions of RT for the right femoral lesser trochanter. During this period, the patient was transferred to a hospice following RT; therefore, treatment effects and imaging evaluations were not available.

Case 2

**Figure 4:**
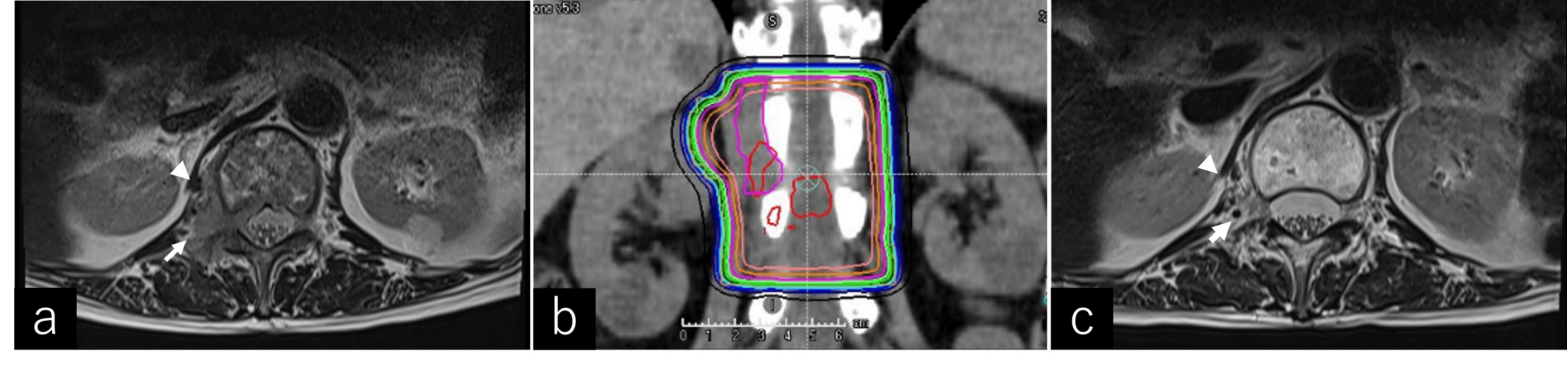
Case 2: MRI and radiation therapy planning for lumbar spine tumor invasion. Magnetic resonance imaging of the lumbar spine revealed tumor invasion of the intervertebral foramen of the 1st lumbar vertebra (arrow), with no evidence of significant invasion into the psoas muscle (arrowhead) (a). RT planning involved irradiation from two fields in the anterior-posterior direction. The patient received 37.5 Gy in 15 fractions of RT for the 1st and 2nd lumbar vertebrae (b). A T2-weighted image obtained 1 month after radiation therapy showed a reduction in tumor size (arrow and arrowhead) (c).

A 72-year-old Japanese woman underwent total mastectomy for breast cancer (cT2aN0M0, stage II) 13 years ago. She had bone metastases in the left seventh rib, 1st lumbar vertebra, 2nd lumbar vertebra, and sacrum, for which she underwent chemotherapy. The patient began experiencing lumbar back pain (Numeric Rating Scale (NRS) 5), discomfort in the right groin area, and difficulties in walking. Although she could still walk, an MRI scan revealed a mass shadow in the intervertebral foramen between the 1st and 2nd lumbar vertebrae (Figure 4a), and the IT sign was positive. The patient received 37.5 Gy in 15 fractions of RT for the 1st and 2nd lumbar vertebrae (Figure 4b). After RT, the patient’s back pain improved to NRS 0-1, and her walking difficulties were alleviated. An MRI scan 6 months post RT revealed that the tumor continued to shrink (Figure 4c).

Case 3

**Figure 5:**
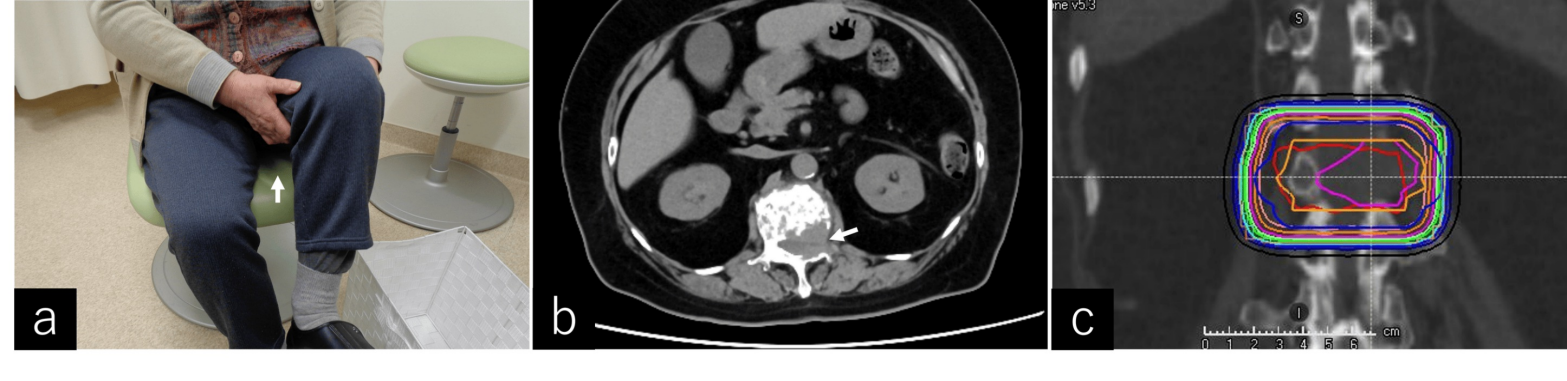
Case 3: Ishikawa-Teramura sign, CT imaging of lumbar spine tumor invasion, and radiation therapy planning. Ishikawa–Teramura Sign (IT sign) (a). CT of the lumbar spine revealed tumor invasion of the 1st lumbar vertebra (arrow) with no evidence of significant invasion into adjacent structures (b). Radiation therapy (RT) planning involved irradiation from two fields in the anterior-posterior direction. The patient received 36 Gy in 12 fractions of RT for the 1st lumbar vertebra (c).

A 75-year-old woman developed low back pain two years post total mastectomy for right breast cancer. A physical examination revealed that the right hip was painful and had a limited range of motion. However, the IT sign was positive (Figure 5a). A CT scan revealed bone metastases in the 7th and 8th thoracic vertebrae, 1st lumbar vertebra (Figure 5b), and right 9th rib. Initially, the patient experienced pain in her right hip due to osteoarthritis, which made walking somewhat uncomfortable. On the left side, although there was no obvious pain in the left groin, the patient had been experiencing frequent tripping over the past few months. Walking required a cane, walker, or wheelchair. The 1st lumbar spine received 36 Gy in 12 fractions of palliative RT (Figure 5c). After treatment, the left hip regained independent mobility, and gait stability was achieved. The need for a walker or cane was eliminated.

MPS

The characteristics of patients with MPS are summarized in Table 2. All patients were treated as inpatients, and no outpatient treatment was administered. Pain control primarily involved the administration of strong opioids (except for one case), and epidural anesthesia was used in two cases. The attending physician accurately diagnosed and intervened in the treatment of MPS in only two cases. It is possible that the other cases were not diagnosed as MPS at the time of treatment. RT was administered as standard palliative RT, with doses ranging from 20 to 30 Gy in most patients (seven patients, 63.7%). One patient using epidural anesthesia completed 54 Gy of irradiation. Treatment efficacy was not clearly described in two patients, but three (27.3%) showed good, mild, and no effect. In two patients, hip flexion became difficult because of atrophy of the psoas major muscle during treatment, which was initially diagnosed as MPS. However, in both cases, gradual psoas atrophy developed over time.

**Table 2:** Patient characteristics of malignant psoas syndrome (n=11). Gy: Gray; IT sign: Ishikawa-Teramura sign; MPS: Malignant psoas syndrome; N: Number; PS: Performance status.

	N (%)/median
Age (years)	61 (range: 40-73)
Sex	
Male	3 (27.3)
Female	8 (27.7)
Primary site and histological type	
Lung cancer	1 (9.1)
Breast cancer	1 (9.1)
Uterine cancer	2 (18.2)
Renal/urologic cancer	2 (18.2)
Gastrointestinal cancer	2 (18.2)
Soft tissue tumor	1 (9.1)
Other	2 (18.2)
Pathology	
Adenocarcinoma	4 (36.3)
Squamous cell carcinoma	1 (9.1)
Transitional cell carcinoma of the urinary tract	1 (9.1)
Renal cell carcinoma	1 (9.1)
Invasive ductal carcinoma	1 (9.1)
Sarcoma	2 (18.2)
Other	1 (9.1)
Imaging	
CT	9 (81.8)
CT and MRI	2 (18.2)
Cause of MPS	
Direct invasion	2 (18.2)
Metastasis	9 (81.8)
Maximum lesion diameter (cm)	9.7 (range; 1.0-14.0)
Left or right	
Left	6 (54.6)
Right	4 (36.3)
Left and right	1 (9.1)
Pain location	
Lower limb	8 (72.7)
Inguinal	1 (9.1)
Hip	1 (9.1)
Lower back	7 (63.6)
Knee	1 (9.1)
Numbness in the lower limb	2 (18.2)
Paralysis in the lower limb	2 (18.2)
Straight leg raise test/psoas stretch test	4 (36.3)
Hip fixed flexion	5 (45.5)
IT sign	1 (9.1)
Gait disorder	10 (90.9)
Walking with a cane	2 (18.2)
Use of a wheelchair	9 (81.8)
PS	
1	1 (9.1)
2	8 (27.7)
3	2 (18.2)
Types of analgesics	
Acetaminophen	3 (27.3)
NSAIDs	4 (36.3)
Weak opioids	1 (9.1)
Strong opioids	11 (100)
Pregabalin	4 (36.3)
Concurrent systemic chemotherapy	1 (9.1)
Recognition of MPS by the attending physician	2 (18.2)
Radiation regimen	
>30 Gy	3 (72.3)
30 Gy	3 (72.3)
20 Gy	4 (36.3)
<20 Gy	1 (9.1)
Dose per fraction	
4 Gy	5 (45.5)
3 Gy	4 (36.3)
2 Gy	1 (9.1)
1.8 Gy	1 (9.1)
Treatment response by radiation therapy	
Good response	3 (27.3)
Mild response	3 (27.3)
Persistence	3 (27.3)
Unclear	2 (18.1)

Survival analysis of all patients

**Figure 6:**
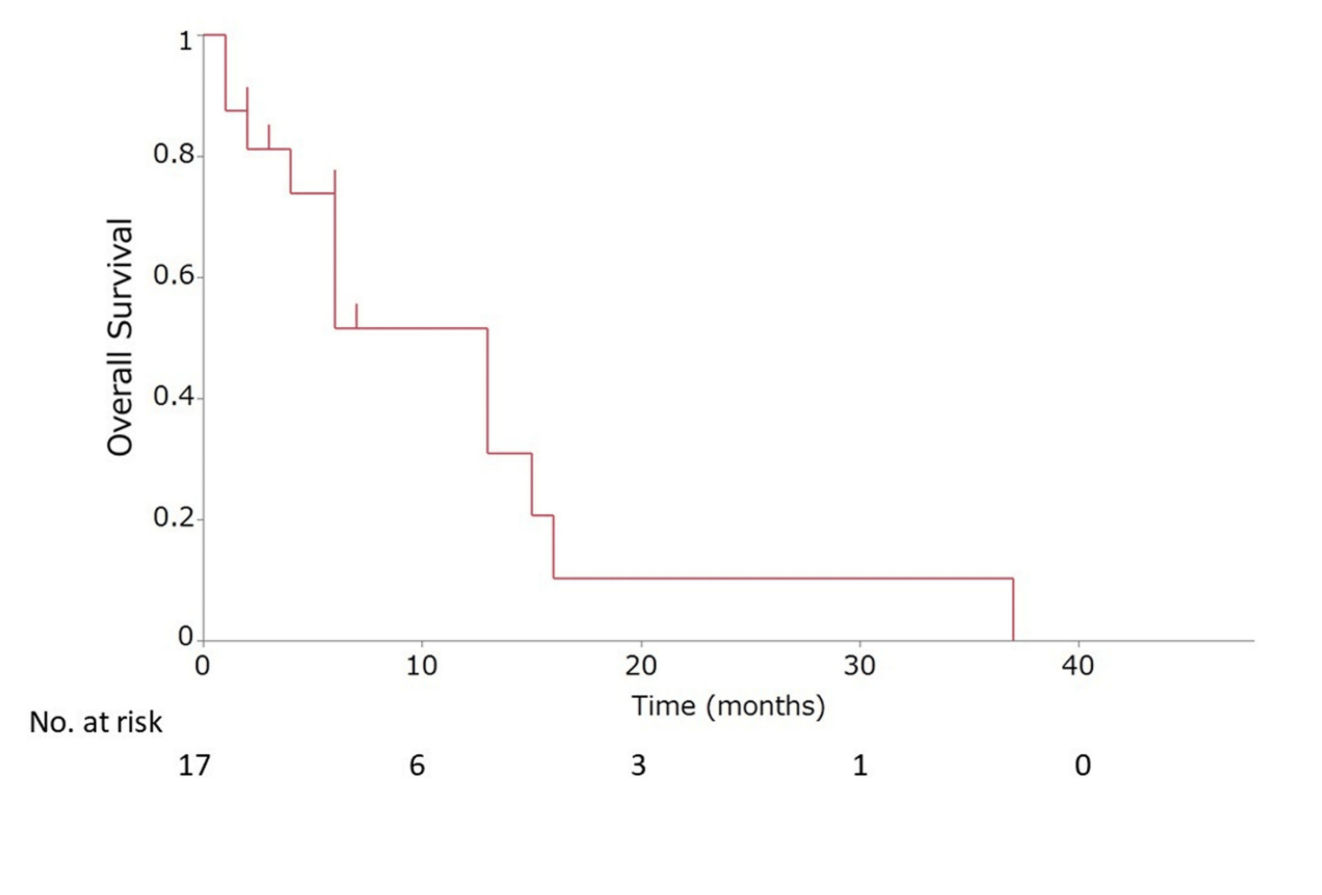
The overall survival rate. The overall survival rate was based on 17 cases.

The survival analysis results for all patients with MPS and MHFFS patients (N = 17) are shown in Figure 6. The median survival time from RT initiation was 13.2 months. Among the MPS and MHFFS patients, five reached termination due to hospice care or transfer to other hospitals, 10 patients died, and two patients are currently surviving. No apparent acute adverse events or late effects resulting from RT have been reported.

## Discussion

MPS, first described in 1990, typically results from distant metastasis of a malignant tumor or direct invasion of the psoas muscle, leading to entrapment of the lumbosacral plexus. This condition manifests as neuropathic pain in the lumbosacral plexus region, primarily during hip extension [1,2,11,3-10]. A similar condition is known as lumbosacral plexopathy [14]. Patients often experience gait disturbances and may require bed rest, or the use of a wheelchair or walker for mobility [11]. Previous reviews and retrospective studies have shown that not all typical symptoms of MPS are present [3,11].

Our study highlights key differences between MPS and MHFFS, particularly in their prevalence, clinical presentation, imaging findings, and response to treatment. MPS was more prevalent (3.8%) compared to MHFFS (2.1%) among the study population, indicating a higher incidence of psoas muscle involvement in metastatic scenarios. Clinically, MPS was characterized by severe neuropathic pain due to direct invasion or compression of the lumbosacral plexus, often leading to significant mobility issues requiring strong opioids or epidural anesthesia for pain management. Imaging findings for MPS typically showed metastatic lesions affecting the lumbosacral plexus region, corroborating the severe pain and neurological symptoms observed in these patients.

Conversely, MHFFS, despite involving the psoas muscle, presented with relatively milder symptoms and was primarily diagnosed based on hip flexion failure rather than severe pain. Imaging findings for MHFFS frequently revealed metastatic lesions at the upper lumbar region or the lesser trochanter of the femur near the psoas muscle's origin or termination. This anatomical difference in tumor location may explain the distinct symptomatology observed in MHFFS patients, who experienced hip flexion failure without the intense neuropathic pain typical of MPS. MHFFS patients showed improvement in hip flexion function with palliative RT, which was not commonly observed in MPS patients. The pain associated with MHFFS was manageable with acetaminophen, NSAIDs, and weak opioids, unlike the intense pain in MPS necessitating stronger analgesics. The distinction in treatment response highlights the importance of tailored therapeutic approaches based on the specific characteristics of each condition.

A recent case of bone metastasis near the femoral obturator area resulting in difficulty in hip flexion with psoas muscle involvement but without painful immobilization of the hip or worsening pain during extension of the lower extremity was described in a recent report [13]. In another case of vertebral metastasis and nerve root involvement near the origin of the psoas muscle, no painful immobilization of the hip or worsening of pain during lower limb extension was reported [12]. Based on these previous reports and our own experience, we propose that tumor invasion at the origin of the psoas muscle, such as the 11th and 12th thoracic vertebrae, as well as the 1st and 2nd lumbar vertebrae, or at the termination of the psoas muscle may be the cause of symptomatic hip flexion failure due to psoas muscle dysfunction. We proposed the term “MHFFS” to distinguish this condition from MPS because the symptoms of this condition differ from those of classic MPS despite tumor extension into the psoas muscle. In our study, we found that the prevalence of MHFFS was approximately 2.1%, which is slightly lower than that of MPS. However, this prevalence is not considered low among radiation oncologists in their daily practice because it reflects the prevalence among patients who receive palliative RT.

It is worth noting that metastatic tumors in anatomically distant sites such as T12, L1, L2, and the lesser trochanter in patients with MHFFS can lead to hip flexion failure, discomfort, and pain around the hip. This finding may be attributed to the distribution of the T12, L1, and L2 sensory nerves near the inguinal region [15]. Previous reports suggest that the lumbar and sacral nerves are frequently affected in the L4-S1 segment (in over 50% of cases), followed by the L1-L4 segment (31%) and radiculopathy (approximately 10%). Plexopathy in the lumbosacral region occurs within one year after diagnosis in more than one-third of patients with primary tumors [14].

**Figure 7:**
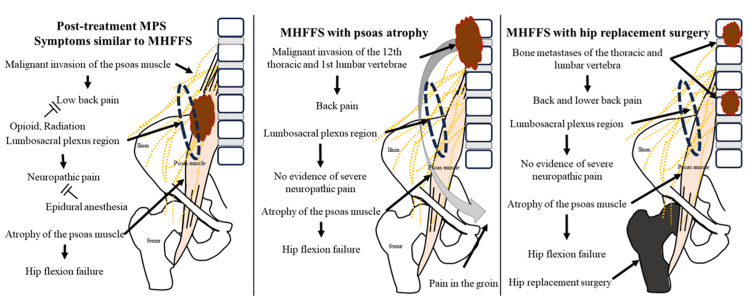
Post-treatment MPS symptoms, MHFFS with psoas major atrophy, and similarities in hip replacement surgery symptoms. Post-treatment MPS symptoms similar to MHFFS include extended management of lumbosacral plexus pain through opioids, epidural anesthesia, and RT, which, along with persistent long-term gait disturbances, can lead to atrophy of the psoas muscle, resulting in clinical manifestations akin to those observed in MHFFS. MHFFS with psoas major atrophy often results from tumors extending to the cephalic end of the psoas muscle, typically near the lower thoracic spine, such as the 12th thoracic vertebra, or the upper lumbar spine, such as the 1st and 2nd lumbar vertebrae. The Ishikawa-Teramura sign (IT sign), described by Teramura S et al. and Ishikawa Y et al., is used to assist hip flexion by lifting the lower extremity with the patient’s hand and is positive [12,13]. Symptoms similar to MHFFS after hip replacement surgery: Conditions that should be differentiated from MHFFS include post-femoral arthroplasty. Bone metastases in the thoracic or lumbar spine accompanied by psoas muscle atrophy following femoral head arthrodesis exhibit clinical symptoms similar to those of MHFFS, and the IT sign may be positive, although the pain is typically not as severe as that in MPS or MHFFS. MPS: Malignant psoas syndrome; MHFFS: Malignant hip flexion failure syndrome. Image credits: Yojiro Ishikawa.

The psoas sign is a medical indicator of psoas muscle irritation. Patients with MPS or psoas abscesses may also present with a positive psoas major sign [11,16]. However, there were no obvious positive cases of the psoas sign in the MHFFS group included in this study. This lack of symptoms can be attributed to the notion that symptoms of irritation to the iliopsoas muscle group are less common in MHFFS than in MPS. Another notable feature of MHFFS is gait disturbance, characterized by lifting the lower leg with both hands due to hip flexion dysfunction (IT sign). However, this is not well-known in daily clinical oncology practice. It should be noted that for an accurate evaluation, the patient must be able to flex the knee joint in the supine position, which is characteristic of MHFFS. Additionally, other muscles of the lower extremity, such as the quadriceps, must also be evaluated. This is crucial because if the knee can not be flexed while in the supine position, it is possible that this condition is not MHFFS but is caused by another condition that results in paralysis of the entire lower extremity. Malignant tumors, particularly metastatic tumors, often cause motor deficits in peripheral nerves [17]. In theory, all nerves and muscles may be affected, but metastasis of the tumor to the muscles is typically minimal [18]. Two patients in the MPS group exhibited gradual loss of psoas muscle function during the disease. This suggests that the progression of MPS may contribute to MHFFS. Given that previous MPS reports featured lower extremity paralysis [3,11], it is conceivable that cases involving psoas muscle dysfunction or atrophy may have been included in those reports. The need to distinguish medically induced psoas muscle atrophy [19], as observed in cases involving femoral artificial joint replacement, is also important. Based on the information presented above, the following figures summarize the cases of hip dysfunction caused by malignancy and their differentiation (Figure 7).

Although the specific tumors at a higher risk of these symptoms are unknown, MPS has been reported more frequently in patients with gynecologic, urologic, and pelvic tumors, including rectal primary tumors or metastases [3,11], whereas MHFFS occurred mainly in patients with bone metastases of hepatocellular carcinoma, breast cancer, or colorectal cancer in this study. The incidence of bone metastasis in patients with hepatocellular carcinoma ranges from 1.97% to 23.59% [20]. Bone metastasis to the lower leg is also relatively rare (5.0%), and metastasis to the lesser trochanter is even rarer [21]. Cases of colorectal cancer metastasizing to the 11th and 12th thoracic, as well as the 1st lumbar vertebrae, have also been reported, but breast cancer was more common in this case series. This may be because breast cancer is a common cause of bone metastasis to the spine [22].

The lumbosacral plexus is situated within the belly of the psoas muscle, and MPS often results in more severe pain. Strong opioids were administered to all patients, and epidural anesthesia was used in some cases. Similar findings have been reported in previous reviews, noting that MHFFS pain is relatively mild and manageable through the use of acetaminophen, non-steroidal anti-inflammatory drugs (NSAIDs), and weak opioids. Previous reports have indicated that pain levels vary between lesions at the attachment of the psoas muscle and those within the muscle belly, with pain levels in the latter typically being more intense [23]. The median survival period for patients with MPS and MHFFS was approximately one year, which is a relatively long survival period for palliative RT in patients with advanced cancer or poor performance status. Alternatively, pain tended to be alleviated easily when radiation was applied to the pain site, and hip flexion function improved in half of the MHFFS patients. In contrast, MPS is characterized by severe pain. Although RT may reduce pain, none of the MPS patients reported improvement in gait disturbance. Because MHFFS is characterized by early improvement after RT, early detection and treatment may help improve patients’ quality of life (QoL). Considering the impact on QoL and prognosis of cancer patients [24], identifying conditions such as MHFFS at an early stage and initiating treatment promptly are crucial to minimize QoL deterioration.

There were several limitations in this study. First, it was a retrospective study. Additionally, because information on clinical findings was determined based on medical records, it was challenging to evaluate cases in which examination findings were not described in detail, and some cases were difficult to evaluate because of transfers to other hospitals. Contrast-enhanced MRI was rarely performed, making it difficult to identify lesions in many cases. While this study provided valuable insights, the lack of neurologists, orthopedic surgeons, and other specialists among the co-authors may have limited the generalizability and the full acceptance of the conclusions drawn. We conducted a survival analysis using a sample size of 17 cases. Previous studies also have attempted to estimate the median survival time with small sample sizes. For instance, Kumar D et al. evaluated the median survival time in patients with small cell carcinoma of the esophagus using a limited number of cases [25], and Liu F et al. applied a similar methodology to 37 cases of gallbladder neuroendocrine carcinoma [26].

## Conclusions

In this study, we newly defined MHFFS as a condition involving either primary or metastatic lesions at the origin or distal end of the psoas muscle, and compared it with MPS. We found MPS to be more painful than MHFFS, requiring higher doses of opioids. However, the use of epidural anesthesia allows the completion of treatment with a radical dose. Additionally, patients with MHFFS exhibited a positive response to the IT sign, a thigh-holding maneuver used to compensate for hip flexion insufficiency. This IT sign is valuable for the early diagnosis of these clinical symptoms.
